# Decreased *Npas4* and *Arc* mRNA Levels in the Hippocampus of Aged Memory‐Impaired Wild‐Type But Not Memory Preserved 11β‐HSD1 Deficient Mice

**DOI:** 10.1111/jne.12339

**Published:** 2016-01-17

**Authors:** J. Qiu, D. R. Dunbar, J. Noble, C. Cairns, R. Carter, V. Kelly, K. E. Chapman, J. R. Seckl, J. L. W. Yau

**Affiliations:** ^1^ BHF Centre for Cardiovascular ScienceUniversity of EdinburghEdinburghUK; ^2^ Centre for Cognitive Ageing and Cognitive EpidemiologyUniversity of EdinburghEdinburghUK; ^3^ Sistemic LtdWest of Scotland Science ParkGlasgowUK

**Keywords:** corticosterone, Y‐maze, spatial memory, microarray, glucocorticoids

## Abstract

Mice deficient in the glucocorticoid‐regenerating enzyme 11β‐HSD1 resist age‐related spatial memory impairment. To investigate the mechanisms and pathways involved, we used microarrays to identify differentially expressed hippocampal genes that associate with cognitive ageing and 11β‐HSD1. Aged wild‐type mice were separated into memory‐impaired and unimpaired relative to young controls according to their performance in the Y‐maze. All individual aged 11β‐HSD1‐deficient mice showed intact spatial memory. The majority of differentially expressed hippocampal genes were increased with ageing (e.g. immune/inflammatory response genes) with no genotype differences. However, the neuronal‐specific transcription factor, *Npas4*, and immediate early gene, *Arc*, were reduced (relative to young) in the hippocampus of memory‐impaired but not unimpaired aged wild‐type or aged 11β‐HSD1‐deficient mice. A quantitative reverse transcriptase‐polymerase chain reaction and *in situ* hybridisation confirmed reduced *Npas4* and *Arc* mRNA expression in memory‐impaired aged wild‐type mice. These findings suggest that 11β‐HSD1 may contribute to the decline in *Npas4* and *Arc* mRNA levels associated with memory impairment during ageing, and that decreased activity of synaptic plasticity pathways involving Npas4 and Arc may, in part, underlie the memory deficits seen in cognitively‐impaired aged wild‐type mice.

Cognitive decline is a prominent feature of normal ageing in humans and rodents. However, large inter‐individual differences exist ranging from little change to mild or severe impairments 1, 2. Glucocorticoids (GCs; largely cortisol in humans, corticosterone in rats and mice), released from the adrenal cortex after stress or diurnal activation of the hypothalamic‐pituitary‐adrenal axis, are implicated in age‐related cognitive impairment. Although short‐term elevated GC levels are generally considered adaptive, prolonged exposure can detrimentally affect brain structure and function, particularly in the hippocampus where they decrease neurogenesis, cause dendritic atrophy and impair memory 3, 4. Hippocampus‐dependent memory impairments are associated with elevated circulating GC levels during ageing in humans and rodents 5, 6, 7.

GCs modulate episodic and working memory processes primarily via activation of two nuclear receptors: the high affinity mineralocorticoid receptors (MR) and low affinity glucocorticoid receptors (GR). Both are ligand‐activated transcription factors and are highly expressed in the hippocampus 8. Before accessing receptors, GCs may be subject to intracellular metabolism. The hippocampus highly expresses 11β‐hydroxysteroid dehydrogenase type 1 (11β‐HSD1), which contributes to intracellular GC levels by catalysing the regeneration of cortisol and corticosterone from inert 11keto forms (cortisone, 11‐dehydrocorticosterone) 9, 10. Our recent studies support a pivotal role for 11β‐HSD1 generated GCs in age‐related cognitive decline 11. Aged 11β‐HSD1 deficient mice show preserved hippocampus‐dependent learning and memory throughout life, resisting age‐related spatial memory deficits observed in wild‐type mice, as shown in both water maze and Y‐maze tasks 12, 13. Conversely, transgenic mice with forebrain specific overexpression of 11β‐HSD1 show accelerated cognitive ageing 14. Short‐term selective inhibition of 11β‐HSD1 reverses spatial memory deficits in aged C57BL/6J mice 15, 16. Improved cognition in aged mice with 11β‐HSD1 deficiency or inhibition associates with reduced intrahippocampal corticosterone (CORT) levels during learning 17, a switch from predominant activation of ‘anti‐cognitive’ GRs to predominant ‘pro‐cognitive’ MRs 18 and enhanced hippocampal long‐term potentiation 13. The downstream genes/pathways beyond receptor activation that underlie age‐related memory deficits associated with 11β‐HSD1 activity are unknown.

Acquisition of long‐term memory requires gene transcription and protein synthesis 19. Gene expression microarrays have identified genes and pathways in rodent hippocampus that associate with cognitive ageing 20, 21, 22, 23. These include down‐regulated immediate early gene (e.g. *Arc*,* Egr‐1*,* Vgf*) and insulin signalling pathways (e.g. *Insr*,* Ide*,* Stat5b*) and up‐regulated general oxidoreductase activity genes (e.g. *Acads*,* Aldh9a1*) selectively in aged cognitively impaired animals 22. To dissect the pathways underlying cognitive protection with 11β‐HSD1 deficiency, the Y‐maze was used to define cognitive function in aged wild‐type and 11β‐HSD1‐deficient mice (relative to young controls) prior to microarray analysis of hippocampal gene transcript profiles. Aged wild‐type mice were further separated into memory impaired and unimpaired groups with the aim of identifying differentially expressed hippocampal genes that associate with cognitive ageing.

## Materials and methods

### Animals

Male mice homozygous for targeted disruption of the *Hsd11b1* gene (*Hsd11b1* ^*−/−*^), congenic on the C57BL/6J genetic background 24, 25, and age‐matched C57BL/6J mice as wild‐type (*Hsd11b1* ^*+/+*^) controls were bred and maintained within our biomedical research facility housed under standard conditions under a 12 : 12 h light/dark cycle (lights on 07.00 h), with food and water *ad lib*. until behavioural testing in the Y‐maze at either 6 months (young) or 24 months (aged). All animal procedures were performed in accordance with the local ethical guidelines of the University of Edinburgh Ethics Committee and those of the UK Animals (Scientific Procedures) Act, 1986.

### Y‐maze

Young (6 months) and aged (24 months) wild‐type and *Hsd11b1* ^*−/−*^ mice were tested in a two trial Y‐maze task previously validated as hippocampus‐dependent 18, 26 for assessment of their spatial memory. All behavioural testing was carried out in the morning (between 08.00 and 11.00 h) in a dimly lit room. Each mouse was placed at the end of one of the three arms of the maze designated the ‘start arm’ and allowed to explore the maze with one arm blocked (novel arm) for 5 min (trial 1) before returning to their home cage. Fixed spatial cues (various objects such as glass bottle, pipette rack, plastic breaker, etc.) surrounded the maze. After an inter‐trial interval (ITI) (either 1 min or 2 h), the mouse was placed back in the maze start arm and allowed to explore all three arms (trial 2) for 5 min 18. The maze was wiped clean with 70% ethanol in between animals to remove olfactory cues. All mice (young, n = 9/genotype; aged wild‐type, n = 13; aged *Hsd11b1* ^*−/−*^ mice, n = 8) were tested first with the 1‐min ITI to ensure they responded to novelty, had no motor deficits and could see the spatial cues around the maze. Spatial memory was tested with the 2‐h ITI, 1 week later. The time spent in each of the arms was calculated with the ANY‐maze software (Stoelting, Dublin, Ireland). Aged mice failing to spend significantly more time in the novel arm compared to the previously visited arms were classed as cognitively impaired, whereas mice showing a preference for the novel arm similar to young controls were cognitively unimpaired. Aged cognitively impaired (AI) and unimpaired (AU) wild‐type mice were randomly selected from the groups for the microarray study (n = 4 per group).

Animals were culled by cervical dislocation between 08.00 and 10.00 h, 3 days after the end of Y‐maze testing. Brains were removed, dissected and the hippocampus was snap frozen in RNase free eppendorf tubes on dry ice and stored at −80 °C. For *in situ* hybridisation studies, brains were frozen on powdered dry ice and stored at −80 °C.

### RNA extraction and processing

Total RNA was extracted from hippocampal tissues of young wild‐type (WT_Y), young *Hsd11b1* ^*−/−*^(KO_Y), aged unimpaired wild‐type (WT_AU), aged impaired wild‐type (WT_AI) and aged *Hsd11b1* ^*−/−*^ (KO_A) mice using TRIzol reagent (Invitrogen, Paisley, UK) and RNeasy Mini Kit (Qiagen, West Sussex, UK). The concentration and purity of each RNA sample was assessed using a GeneQuant RNA/DNA calculator (GE Healthcare, Little Chalfont, UK). Hippocampal RNA samples were processed through standard Affymetrix protocols, and hybridised to Affymetrix Mouse Genome 430 2.0 GeneChips (n = 4 per group; Affymetrix, Santa Clara, CA, USA). CEL files for all 20 chips were imported into the Affy package of bioconductor, and were processed (background subtraction and normalisation) with the robust multichip average algorithm. Chip data quality control was performed by: (i) visual inspection of the chip images (not shown) that showed no obvious abnormalities and (ii) histogram of raw intensities from all chips that showed no clear outliers and had the usual distribution. Quality control indicated that the data were technically good. Expression levels for each chip and fold changes between genotype for each tissue were calculated. Genes with no or very low expression (i.e. expressed below a normalised expression value of 100 in all, or all but one, of the samples) were excluded. Data were exported in text format and imported into a mysql database (https://www.mysql.com). Annotation data for the genes were obtained from netaffx (Affymetrix). A web accessible front‐end query tool was built that allows query of the data by expression data (normalised expression, fold‐changes, P‐values) and by sequence annotation (gene title and symbol, Entrez gene ID, Affymetrix ID and Gene Ontology data). Microarray data are available in the Gene Expression Omnibus (http://www.ncbi.nlm.nih.gov/geo) with accession number GSE68515. Microarray processing was carried out by ARK‐Genomics (Roslin Institute, Edinburgh UK).

### Real‐time quantitative reverse transcriptase‐polymerase chain reaction (RT‐PCR)

Total RNA (1.5 μg) from hippocampal samples from the experimental groups (WT_Y, KO_Y, WT_AU, WT_AI and KO_A) (n = 5–8 per group including overlap with animals used in microarray) was reverse transcribed into cDNA with oligo(dT) primers using the QuantiTect Reverse Transcription Kit (Qiagen) in accordance with the manufacturer's instructions. Gene‐specific mRNA levels were determined in cDNA samples incubated in triplicate with gene‐specific primers and fluorescent probes (using pre‐designed assays from Applied Biosytems, Warrington, UK) in a 1 × Roche LightCyclerR 480 Probes mastermix (Roche, Basel, Switzerland). PCR cycling and detection of fluorescent signal were carried out using a Roche LightCyclerR 480 (Roche). A standard curve was constructed for each primer probe set using a serial dilution of cDNA pooled from all samples. The primers (Invitrogen) were designed for use with intron‐spanning probes from the Roche Universal Probe Library. Software provided with the LightcyclerR 480 was used to analyse the data produced after RT‐PCR. All results were corrected by normalisation to the expression level of the reference (housekeeping) gene *Gapdh*, which did not differ between groups and expressed arbitrarily as an adjusted ratio.

### *In situ* hybridisation

PCR products corresponding to nucleotides 1859–2349 of the mouse *Arc* cDNA and 1055–1607 of the mouse *Npas4* cDNA were generated from control C57BL/6J mouse hippocampus cDNA and subcloned into the pGEM‐T Easy vector (Promega, Madison, WI, USA). ^35^S‐UTP (Perkin Elmer, Waltham, MA, USA) labelled sense and antisense cRNA probes were generated using restriction enzyme‐linearised plasmid as template for *in vitro* transcription using either T7 or SP6 RNA polymerase as appropriate. Cryostat coronal brain sections at the level of the dorsal hippocampus from young and aged WT_AI (WT_AU were omitted as a result of the small sample size) and *Hsd11b1* ^*−/−*^ (KO) mice (n = 6–9 per group, including overlap with animals used in microarray) were post‐fixed in 4% paraformaldehyde, acetylated (0.25% acetic anhydride in 0.1m triethanolamine, pH 8.0), washed in phosphate‐buffered saline, dehydrated in graded ethanol and air‐dried. Sections were hybridised with probe overnight at 50 °C, followed by RNaseA treatment and standard saline citrate buffer washes. Slides were dehydrated in graded ethanol and air‐dried before exposure to Biomax MR‐1 film (Amersham, Little Chalfont, UK) for 5–8 days at room temperature. The slides were then dipped in NTB‐2 emulsion (Eastman Kodak Co, Rochester, NY, USA) and exposed for 3 weeks at 4 °C before developing and counterstaining with 1% pyronine. The hybridisation signal was assessed by computer‐assisted grain counting using ks300 image analysis software (Carl Zeiss, Oberkochen, Germany). Silver grains were counted within a fixed circular area under bright‐field illumination using the × 40 magnification objective, over individual cells by an investigator who was blinded to age and genotype. For each animal, 15–18 cells per subregion of hippocampus or cortical layer were assessed and background, counted over areas of white matter, was subtracted. The labelled RNA probes (antisense and sense) were first hybridised onto control brain sections from adult mice to test their specificity at the autoradiographic level. No binding was detected with labelled sense probes of Npas4 and Arc (data not shown).

### Statistical analysis

Statistical analysis of the microarray gene expression data was carried using limma r/bioconductor 27 yielding multiple testing corrected P‐values for each comparison of interest. A nonparametric statistical test (rank products; RP) was also used. The RP approach has been shown to be reliable for identifying biologically relevant expression differences, even with highly noisy data 28, 29. Genes were considered differentially expressed between groups when rank product P < 0.05. The Y‐maze (2‐h ITI time spent in novel arm) and *in situ* gene expression data across the groups were analysed using two‐way anova with age and genotype as the independent variables followed by Tukey's multiple comparison's test as appropriate for between group comparisons. Comparisons of time spent in the arms of the Y‐maze within each group were analysed by Student's paired t‐tests. The quantitative RT‐PCR data with the aged impaired and unimpaired wild‐type mice as separate groups were analysed by one‐way anova with Tukey's multiple comparison's test as appropriate for between group comparisons. Data are reported as the mean ± SEM.

## Results

### Spatial memory status of aged wild‐type and 11β‐HSD1‐deficient mice in the Y‐maze

**Figure 1:**
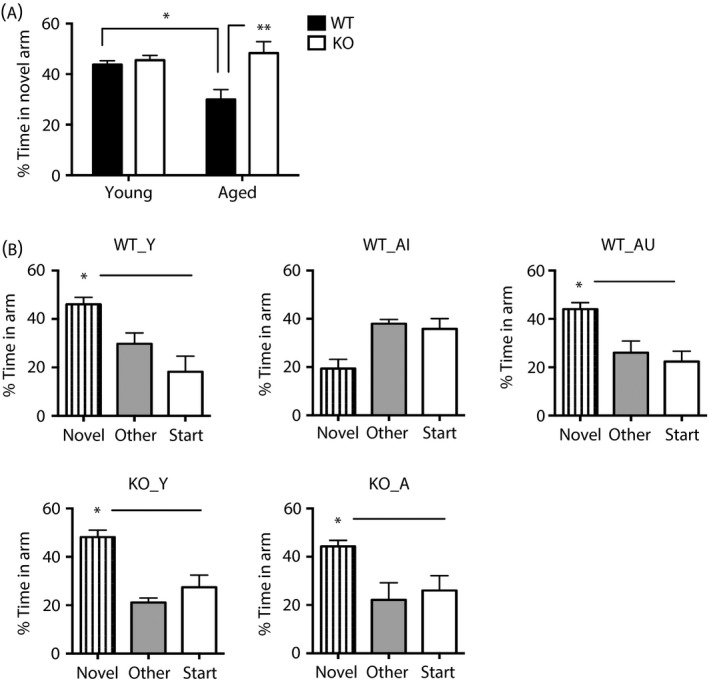
Spatial memory status of aged wild‐type and 11β‐HSD1‐deficient mice in the Y‐maze. (a) Aged (24 months) wild‐type (WT) mice as a group (n = 13) showed impaired spatial memory retention in the Y‐maze after a 2‐h inter‐trial interval (ITI) (less time in novel arm) compared to young (6 months) WT controls (n = 9) and aged (24 months) *Hsd11b1* ^*−/−*^ (KO) mice (n = 8). (b) Spatial memory of mice selected for microarray analysis from (a) showing the five groups used: WT_Y (young wild‐type), KO_Y (young *Hsd11b1* ^*−/−*^), KO_A (aged *Hsd11b1* ^*−/−*^), WT_AI (aged wild‐type memory‐impaired) and WT_AU (aged wild‐type memory‐unimpaired) (n = 4 per group). *P < 0.05, **P < 0.01 significant difference between comparisons. Data shown are the mean ± SEM.

All young and aged wild‐type and *Hsd11b1* ^*−/−*^ mice spent significantly more time in the novel arm than previously visited arms of the Y‐maze (P < 0.01, paired t‐tests) after a 1‐min ITI (see Supporting information, Fig. S1), confirming the aged mice had no impairment of vision or motor deficits. There were little inter‐individual differences within the aged mice groups (both wild‐type and *Hsd11b1* ^*−/−*^) after the 1‐min ITI, with all mice performing equally well regardless of their later cognitive status after the 2‐h ITI. Analysis of the 2‐h ITI Y‐maze data, as a measure of spatial memory, revealed a significant effect of genotype (F_1,33_ = 8.4, P < 0.01) and genotype × age interaction (F_1,33_ = 5.7, P < 0.05) (Fig. 1 a). Aged (24 months) wild‐type mice spent less time in the novel arm during the retention trial after a 2‐h ITI compared to young (6 months) wild‐type controls (P < 0.05) and to aged *Hsd11b1* ^*−/−*^ mice (P < 0.01) (Fig. 1 a), recapitulating previous findings 13, 18. Aged wild‐type mice (n = 13) showed an overall impairment in spatial memory (Fig. 1 a), although examination of individual animals revealed four unimpaired mice with intact spatial memory similar to young mice, spending significantly more time in the novel arm (P < 0.05) than previously visited arms of Y‐maze (Fig. 1 b). Aged memory‐unimpaired wild‐type (WT_AU) mice spent a significantly (P < 0.001) longer percentage time in the novel arm than aged memory‐impaired wild‐type mice (WT_AI) (Fig. 1 b). Mice from each age and genotype group were used for microarray analysis of hippocampal gene expression (Fig. 1 b).

### Differentially expressed hippocampal genes associated with 11β‐HSD1 deficiency, memory impairment and ageing

**Figure 2:**
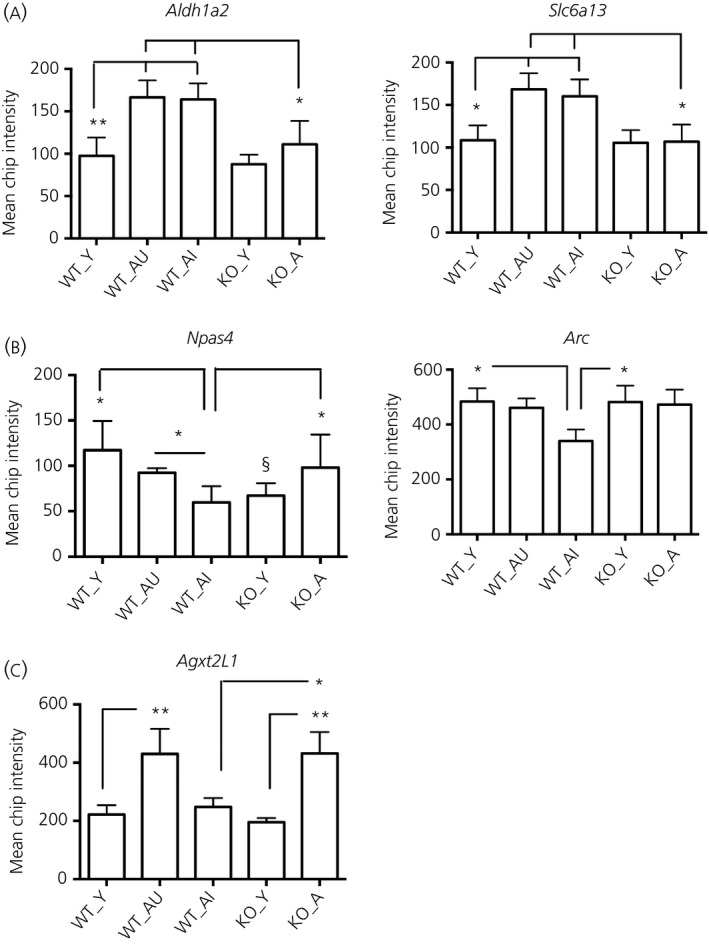
Microarray mean chip intensity of a selection of differentially expressed hippocampal genes in wild‐type and 11β‐HSD1‐deficient mice. (a) Increased with age in WT but not in KO. (b) Decreased with age in WT_AI but not in KO. (c) Increase with age in WT_AU and in KO but not in WT_AI. Comparisons between young and aged wild‐type unimpaired and impaired mice [(WT_Y) and (WT_AU) or (WT_AI), n = 4 per group] and between aged wild‐type and *Hsd11b1* ^−/−^ mice [WT_AI and KO_A, n = 4 per group] differed by approximately 1.5‐fold (*P < 0.05, **P < 0.01 RP score). §P < 0.05 compared to WT_Y. Data shown are the mean ± SEM.

**Figure 3:**
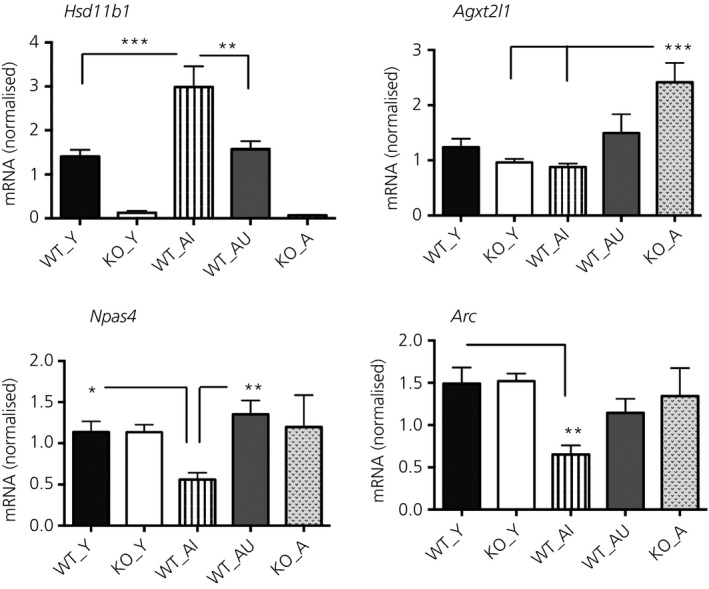
Quantitative real‐time polymerase chain reaction measurement of *Hsd11b1*,* Agxt2l1*,* Npas4* and *Arc* mRNA levels in the hippocampus of wild‐type and 11β‐HSD1‐deficient mice. Levels of *Hsd11b1*,* Agxt2l1*,* Npas4* and *Arc* mRNA in the hippocampus of young wild‐type (WT_Y), young *Hsd11b1* ^*−/−*^ (KO_Y), aged wild‐type impaired (WT_AI), aged wild‐type unimpaired (WT_AU) and aged *Hsd11b1* ^*−/−*^ mice (KO_A) were measured relative to *Gapdh* and expressed as a ratio (n = 5–8 per group). *P < 0.05, **P < 0.01, ***P < 0.001 significance difference between groups. Data shown are the mean ± SEM.

Data from aged wild‐type mice were examined as two subgroups [aged memory‐impaired (WT_AI, n = 4) and aged memory unimpaired (WT_AU, n = 4)] and also as a combined group (WT_A, n = 8). These were compared with aged *Hsd11b1* ^*−/−*^ mice, which showed no cognitive impairment. Most differentially expressed hippocampal transcripts were increased with age but did not differ between genotypes (an approximate 1.5‐fold increase, < 0.05 RP score compared to corresponding young controls). These include inflammatory/immune response genes (*C1qb*,* C1qbc*,* B2m*,* Aif1*,* Fcgr2b*,* Fcgr3*,* Trem2*,* Lyz2*,* Mpeg1*) and glial/structural genes (*Gfap*,* Vim*,* Dmp1*), as well as those for cholesterol/lipid metabolism (*Apod*), signal transduction/transport (*Anxa3*,* Cyba*,* Abca8a*) and protein binding (*Rtp4*,* Tyrobp*) (see Supporting information, Table S1). Some genes were increased with age (approximately 1.5‐fold, P < 0.05 RP score) in wild‐type mice regardless of cognitive status (WT_A) but not in *Hsd11b1* ^*−/−*^ mice, including the rate‐limiting retinoic acid‐synthesising enzyme (*Aldh1a2*) and the GABA transporter 2 (*Slc6a13*) (Fig. 2). Only three genes were differentially expressed significantly between WT_AI and WT_AU (Fig. 2; see also Supporting information, Fig. S2) and between WT_AI and KO_A (Fig. 2; see also Supporting information, Fig. S3). Of these, the transcription factor *Npas4* and immediate early gene *Arc*, are of particular interest because of their link with learning and memory 30, 31. *Npas4* and *Arc* mRNA levels were selectively decreased with age in the hippocampus of WT_AI (P < 0.01 and P < 0.05, RP score, respectively) but not WT_AU or KO_A mice (Figs 2 and 3). *Npas4* mRNA levels were also lower in KO_Y compared to WT_Y (P < 0.05) (Fig. 2). Interestingly, the level of *Agxt2l1* (alanine‐glyoxylate aminotransferase 2‐like 1), a gene whose function is poorly characterised but for which a dysregulation in prefrontal cortex has been associated with mood disorders 32, differed significantly between WT_AU and WT_AI mice (P < 0.01, RP score) and was increased with age in the hippocampus of both WT_AU and KO_A (P < 0.001, RP score) but not WT_AI mice (Figs 2 and 3).

### Decreased *Npas4* and *Arc* but increased *Hsd11b1* mRNA levels in the hippocampus of aged memory‐impaired wild‐type mice

Differential expression of *Npas4* and *Arc* mRNA levels was validated by quantitative real‐time PCR (qPCR) using total RNA from the hippocampus of WT_AI, WT_AU, KO_A and corresponding young mice of each genotype (n = 5–8 per group). However, the microarray changes in *Agxt2l1* mRNA levels were not fully validated by qPCR, with no significant increase in WT_AU compared to WT_AI (P = 0.11) or compared to WT_Y (P = 0.47) (Fig. 3). Although the microarray data analysis did not reveal differences in *Hsd11b1* mRNA levels between young and aged wild‐type mice, qPCR showed significantly higher levels of *Hsd11b1* mRNA in the WT_AI group compared to WT_Y controls (P < 0.001) (Fig. 3) confirming our previous findings 14. Both hippocampal *Npas4* and *Arc* mRNA levels differed significantly between the groups (F_4,27_ = 5.1, P < 0.01 and F_4,26_ = 5.9, P < 0.01, respectively) (Fig. 3). Hippocampal *Npas4* and *Arc* mRNA levels were decreased selectively in WT_AI but not WT_AU mice (WT_Y versus WT_AI, P < 0.05 and P < 0.01, respectively) (Fig. 3). *Npas4* mRNA levels were lower in the hippocampus of WT_AI compared to WT_AU mice (P < 0.01) (Fig. 3). By contrast, both hippocampal *Npas4* and *Arc* mRNA levels were not significantly altered with age in *Hsd11b1* ^*−/−*^ mice (Fig. 3). However, the lower Npas4 mRNA levels in KO_Y versus WT_Y from the microarray data (n = 4/genotype) were not evident by qPCR in the larger sample size (n = 8 per genotype) (Fig. 3).

### Decreased *Npas4* and *Arc* mRNA expression selectively in hippocampal CA1 cells of aged memory‐impaired wild‐type but not 11β‐HSD1‐deficient mice

**Figure 4:**
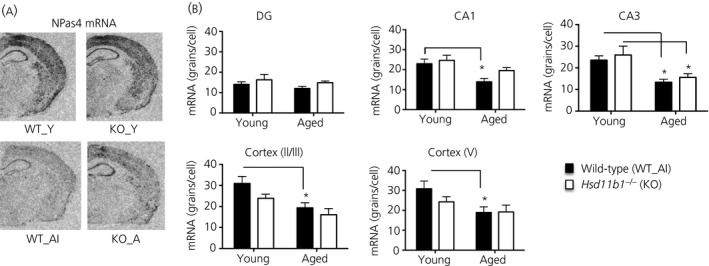
Differentially expressed *Npas4* mRNA in hippocampus and cortex of aged wild‐type and 11β‐HSD1‐deficient mice. (a) Representative *in situ* hydridisation autoradiograms showing *Npas4* mRNA expression in coronal mouse brain sections at the level of the dorsal hippocampus from young wild‐type (WT_Y), young *Hsd11b1* ^*−/−*^ (KO_Y), aged memory‐impaired wild‐type (WT_AI) and aged *Hsd11b1* ^*−/−*^ mice (KO_A) (n = 6–8 per group). Mice were previously tested in the Y‐maze to confirm spatial memory status as in Fig. 1 with only WT_AI mice included for *in situ* hybridisation analysis. (b) Quantification of *Npas4* mRNA levels in hippocampus subregions (dentate gyrus, DG, CA1 and CA3) and cortex (layers 2/3 and V) of wild‐type and *Hsd11b1* ^*−/−*^ mice. *P < 0.05 significant difference between groups. Data shown are the mean ± SEM.

We performed *in situ* hybridisation to gain a regional resolution of the reduced hippocampal *Npas4* and *Arc* mRNA expression in aged memory‐impaired wild‐type mice. In young wild‐type mice, *Npas4* mRNA expression was greatest in the cortical region (layers 2/3 and 5) and hippocampus, particularly in the CA1 and CA3 cell layers (Fig. 4 a). Within the CA1 subregion of the hippocampus, *Npas4* mRNA levels were decreased with age (approximate 40% reduction, F_1,26_ = 11.8, P < 0.01) in WT_AI but not KO_A mice (Fig. 4 b). Levels of *Npas4* mRNA in the CA1 region showed a nonsignificant trend to be lower in WT_AI mice compared to KO_A mice (F_1,26_ = 3.2, P = 0.08) (Fig. 4 b). CA3 *Npas4* mRNA levels were decreased with age regardless of genotype (approximate 40% reduction, F_1,24_ = 19, P < 0.001) (Fig. 4 b). In the cortex, levels of *Npas4* mRNA were affected by age but not genotype (cortical layer 2/3, F_1,25_ = 12.9, P < 0.01; cortical layer 5, F_1,25_ = 6.8, P < 0.05) (Fig. 4 b); post‐hoc analysis showed a decrease with age in WT_AI (approximate 37% decrease, P < 0.05) but not KO_A mice (Fig. 4 b).

**Figure 5:**
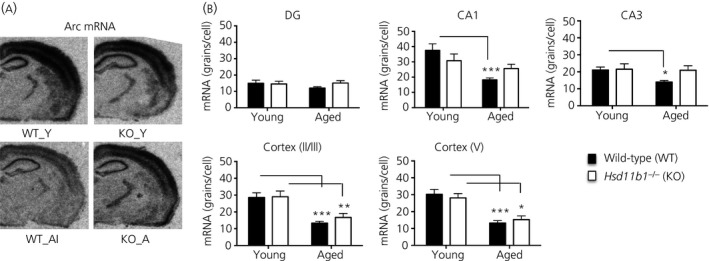
Differentially expressed *Arc* mRNA in hippocampus and cortex of aged wild‐type and *11*β*‐HSD1‐deficient* mice. (a) Representative *in situ* hybridisation autoradiograms showing *Arc* mRNA expression in coronal mouse brain sections at the level of the dorsal hippocampus from young wild‐type (WT_Y), young *Hsd11b1* ^*−/−*^ (KO_Y), aged memory‐impaired wild‐type (WT_AI) and aged *Hsd11b1* ^*−/−*^ mice (KO_A) (n = 6–9 per group). Mice were previously tested in the Y‐maze to confirm spatial memory status as in Fig. 1 with only WT_AI mice included for *in situ* hybridisation analysis. (b) Quantification of *Arc* mRNA levels in hippocampus subregions (dentate gyrus, DG, CA1 and CA3) and cortex (layers 2/3 and V) of wild‐type (WT) and *Hsd11b1* ^*−/−*^ mice. *P < 0.05, **P < 0.01, ***P < 0.001 significant difference between groups. Data shown are the mean ± SEM.

Levels of *Arc* mRNA were highest in the CA1 cell layer of the hippocampus and layers 2/3 and 5 of the cortex (Fig. 5 a). In the hippocampus, there was an age (F_1,27_ = 14.8, P < 0.001) and age × genotype interaction (F_1,27_ = 5.0, P < 0.05) selectively in CA1 (Fig. 5 b). *Arc* mRNA levels were decreased with age in hippocampal CA1 cells of WT_AI mice (approximate 50% reduction, P < 0.001) and CA3 (approximate 33% reduction, P < 0.05) but not KO_A mice (Fig. 5 b). In the cortex, *Arc* mRNA levels were decreased with age but not genotype (cortical layer 2/3, F_1,30_ = 32, P < 0.001; cortical layer 5, F_1,30_ = 40, P < 0.001) (Fig. 5 b). Post‐hoc analysis revealed a decrease with age in both WT_AI (approximate 52–57% decrease, P < 0.001) and KO_A mice (approximate 41–46% decrease, P < 0.05) (Fig. 5 b).

## Discussion

Lifelong deficiency or short‐term inhibition of 11β‐HSD1 consistently preserves or improves spatial memory in aged mice 12, 13, 15, 18. In the present study, we identified two hippocampal genes, the brain‐specific activity‐dependent transcription factor *Npas4* (neuronal Per‐Arnt‐Sim domain protein 4) and the immediate early gene *Arc* (activity‐regulated cytoskeleton‐associated protein), as being differentially expressed with ageing, cognitive decline and 11β‐HSD1 deficiency. Given the crucial roles of Npas4 and Arc in the regulation of learning and memory 31, 33, 34, 35, 36, 37, and their decreased mRNA levels in the hippocampus of aged memory‐impaired (AI) but not unimpaired (AU) wild‐type or 11β‐HSD1‐deficent mice, it is suggested that these proteins may lie in pathways that are important for the preservation of hippocampus‐associated memory in ageing and are maintained by 11β‐HSD1 deficiency/inhibition.

Several studies have used microarrays to identify hippocampal gene transcripts associated with cognitive ageing under basal (home cage) and memory‐activated (1 h after water maze training) conditions in rats 22, 23, 38, 39, 40 and mice 41, 42. The number of hippocampal genes identified as differentially expressed between aged cognitively impaired and unimpaired animals vary, with some studies revealing more genes altered than others. Importantly, several of the transcripts elevated with ageing regardless of genotype or cognitive status in the present study were also identified as genes regulated by ageing and not memory decline in cognitively tested aged rats, including inflammatory/immunity genes (*C1qc*,* C1qb*,* B2m*,* Aif1*,* Fcgr2b*) and structural genes (*Gfap*,* Vim*), as well as genes for cholesterol/lipid metabolism (*Apod*) and signal transduction (*Anxa3*) 21, 22, 43. This affords some confidence that these reflect ageing *per se* rather than the processes underlying cognitive variation with age.

By contrast to previous studies in rats that examined memory‐activated hippocampal gene expression profiles 22, 23, only three notable hippocampal genes were differentially expressed between the AI and AU wild‐type mice as characterised in the Y‐maze spatial recognition memory task. This low number of differentially expressed hippocampal genes suggests that the AI and AU characterisation based on a single ‘non‐aversive’ Y‐maze trial may not be as reliable as previous methods that use multiple ‘aversive’ water maze trials as shown in aged mice 42 and rats 6, 44, 45. The AU wild‐type mice would benefit from further characterisation in the water maze to demonstrate a consistent phenotype of preserved memory function. Thus, the implications of the altered gene transcript levels in AU wild‐type mice are less clear and could simply reflect within‐subject variability rather than a consistent distinct subject cognitive phenotype. By contrast, the impaired and preserved cognitive phenotype of the key comparisons between AI wild‐type and aged 11β‐HSD1‐deficient mice, respectively, have been reliably confirmed in previous studies following both Y‐maze and water maze protocols 12, 13, 18. Among the differentially expressed genes, *Arc* transcript levels were reduced selectively in AI wild‐type mice, a finding consistent with the correlation of hippocampal *Arc* mRNA levels with spatial memory 46 and previous studies in aged rats with memory deficits 21, 22, 47, 48. Acutely, both a memory‐enhancing dose of CORT and learning have been shown to increase *Arc* mRNA and protein expression 43, 46, 49 indicating a plausible pathway linking stress and its GC mediators through life, cognition and individual differences in cognitive decline with age. Moreover, hippocampal *Arc* expression was reduced in CA1 and CA3 but not the dentate gyrus of AI wild‐type mice in line with subregional changes in basal levels of *Arc* mRNA in aged rats 47. Decreased *Arc* mRNA levels have been reported during both ‘offline’ periods of rest and following spatial behaviour in the aged hippocampus 47. Indeed, *Arc* mRNA levels under resting home cage conditions are considered to reflect active information processing in cells that previously transcribed *Arc* in response to behaviour 50. Thus, reduced levels of *Arc* mRNA in CA1 and CA3 pyramidal cells of AI wild‐type mice may reflect impaired memory consolidation, as in *Arc* ^−/−^ mice 51, during the rest (home cage) period. Importantly, aged 11β‐HSD1‐deficient mice and AU wild‐type mice do not show reduced *Arc* mRNA levels in CA1 and CA3 cells and are not impaired in the Y‐maze.

*Npas4* mRNA levels were also decreased in the hippocampal CA1 region of AI wild‐type but not aged 11β‐HSD1‐deficient mice. Reduced levels of *Npas4* mRNA in hippocampus have previously been noted in aged rats 52, although this has not been specifically associated with the subset of animals showing cognitive decline. However, recent evidence indicates a key role for Npas4 in memory formation 33, 34. Moreover, Npas4 influences the survival of neurones, development and the maintenance of synapses, as well as the regulation of synaptic plasticity 30, via downstream target genes, including brain‐derived neurotrophic factor 53. Indeed, in a separate study, we found hippocampal levels of *Bdnf* mRNA to be reduced in aged wild‐type but not aged 11β‐HSD1‐deficient mice (S. Caughey, A.P. Harris, J.R. Seckl, M.C. Holmes and J.L.W. Yau, unpublished data). This is consistent with the lower *Npas4* mRNA levels in AI wild‐type mice, decreased mRNA levels of both *Bdnf* and *Npas4* in the hippocampus of aged rats 52, and reduced transcription of multiple *Bdnf* isoforms in *Npas4* ^−/−^ mice 54.

Putative negative glucocorticoid response elements (GREs) found upstream of the *Npas4* transcription initiation site suggest regulation by CORT 55. Indeed, *in vivo* treatment with high CORT doses reduce *Npas4* mRNA and protein expression in the mouse hippocampus and frontal cortex 55, 56. This suggests that the maintained *NPas4* mRNA levels in AU wild‐type mice and aged 11β‐HSD1‐deficient mice may, at least in part, be a result of lower brain intracellular CORT levels as a consequence of decreased 11β‐HSD1 activity 17. In support of this notion, hippocampal *Hsd11b1* mRNA levels were selectively increased in AI but not AU wild‐type mice compared to young wild‐type mice.

It is possible that the greater rise in hippocampal CORT levels induced by learning/training in aged wild‐type mice 17 activates GRs, which in turn reduce *Npas4* transcription directly by binding to negative GREs in its promoter 55. A decrease in *Npas4* mRNA expression may contribute to reduced transcription of its target gene, *Bdnf* 52, which regulates neuroplasticity and memory mechanisms 30, 52. Lower *Npas4* expression may also affect the expression of *Arc* indirectly. Indeed, memory‐activated expression of *Npas4 mRNA* in the dorsal hippocampus of mice appears upstream of several other immediate‐early genes including *Arc* 33. Moreover, conditional deletion of *Npas4* in cultured mouse hippocampal neurones abolished the depolarisation‐induced expression of *Arc* mRNA 33. Thus, a reduced *Npas4* expression could contribute to lower levels of *Arc* transcripts in the hippocampus of AI wild‐type mice.

These findings implicate both Npas4 and Arc in the pathways that may underlie the impairment and maintenance of spatial memory associated with ageing and 11β‐HSD1 deficiency. However, any causal relationship between 11β‐HSD1 deficiency, resistance to ageing‐associated decline of *Npas4* and *Arc* mRNA levels, and ageing‐associated spatial memory deficits remains to be determined. If 11β‐HSD1 deficiency causes resistance to age‐related decline of hippocampal *Npas4* and/or *Arc* mRNA levels, then short‐term selective inhibition of 11β‐HSD1 in aged C57BL/6J mice, which reverses spatial memory impairments 16, would be anticipated to associate with increased *Npas4* and/or *Arc* mRNA levels. Future studies could examine this in aged mice during 11β‐HSD1 inhibitor treatment when spatial memory is improved, and after stopping treatment when memory reverts back to impaired, to test whether *Npas4* and/or *Arc* mRNA levels are increased and decreased, respectively. Furthermore, intrahippocampal administration of high CORT levels to aged 11β‐HSD1 deficient mice (i.e. to levels equivalent to those found in aged wild‐type mice) could be carried out to establish whether it is the lower hippocampal CORT levels as a consequence of 11β‐HSD1 deficiency 17 that prevent the decreased *Npas4* and *Arc* mRNA levels and/or memory deficits. It is likely that there are other hippocampal synaptic plasticity genes modulated by 11β‐HSD1 activity playing a role in the variable cognitive decline with ageing. Examination of learning‐activated gene transcripts and proteins in the hippocampus and functional characterisation of selected genes *in vivo* could help provide further insight into the mechanisms whereby 11β‐HSD1 activity contributes to age‐related memory decline.

## Supporting information


**Figure S1.** Initial assessment of young and aged wild‐type and 11β‐HSD1‐deficient mice in the Y‐maze following a 1‐min inter‐trial interval (ITI).
**Figure S2.** Comparison of scatter plots of log intensity values (four replicates combined) for young and aged wild‐type mice main comparisons.
**Figure S3.** Comparison of scatter plots of log intensity values (four replicates combined) for young and aged wild‐type and 11β‐HSD1‐deficient mice main comparisons.
**Table S1.** Hippocampal genes up‐regulated with ageing in wild‐type and 11β‐HSD1‐deficient mice but not affected by genotype.
