# Supplementary material for: Decreased Npas4 and Arc mRNA Levels in the Hippocampus of Aged Memory‐Impaired Wild‐Type But Not Memory Preserved 11β‐HSD1 Deficient Mice
Source: J Neuroendocrinol. 2016 Jan 17;28(1):n/a. doi: 10.1111/jne.12339 (PMC4737280; doi:10.1111/jne.12339)
Supplement: Supplementary file 1 — Figure S1. Initial assessment of young and aged wild‐type and 11β‐HSD1‐deficient mice in the Y‐maze following a 1‐min inter‐trial interval (ITI). Figure S2. Comparison of scatter plots of log intensity values (four replicates combined) for young and aged wild‐type mice main comparisons. Figure S3. Comparison of scatter plots of log intensity values (four replicates combined) for young and aged wild‐type and 11β‐HSD1‐deficient mice main comparisons. Table S1. Hippocampal genes up‐regulated with ageing in wild‐type and 11β‐HSD1‐deficient mice but not affected by genotype. [file JNE-28-0-s001.pdf]

Supplementary Information

Decreased *Npas4* and *Arc* mRNA levels in the hippocampus of aged memory-impaired wild type but not memory preserved 11β-HSD1 deficient mice

Jing Qiu [a], Donald R. Dunbar [a, l], June Noble [a], Carolynn Cairns [a], Rod Carter [a], Val Kelly [a], Karen E. Chapman [a], Jonathan R. Seckl [a, b], Joyce L.W. Yau [a, b, *]

[a]BHF Centre for Cardiovascular Science, University of Edinburgh, UK
[b]Centre for Cognitive Ageing and Cognitive Epidemiology, University of Edinburgh, UK

* Corresponding author:
Dr Joyce LW Yau**,**
BHF Centre for Cardiovascular Science,
The Queen's Medical Research Institute,
47 Little France Crescent, Edinburgh EH16 4TJ, UK.
Tel: +44 (0) 131 242 6760
Fax: +44 (0) 131 242 6779
E-mail: Joyce.Yau@ed.ac.uk

[l] Present address: Sistemic Ltd, West of Scotland Science Park, Glasgow G20 OSP

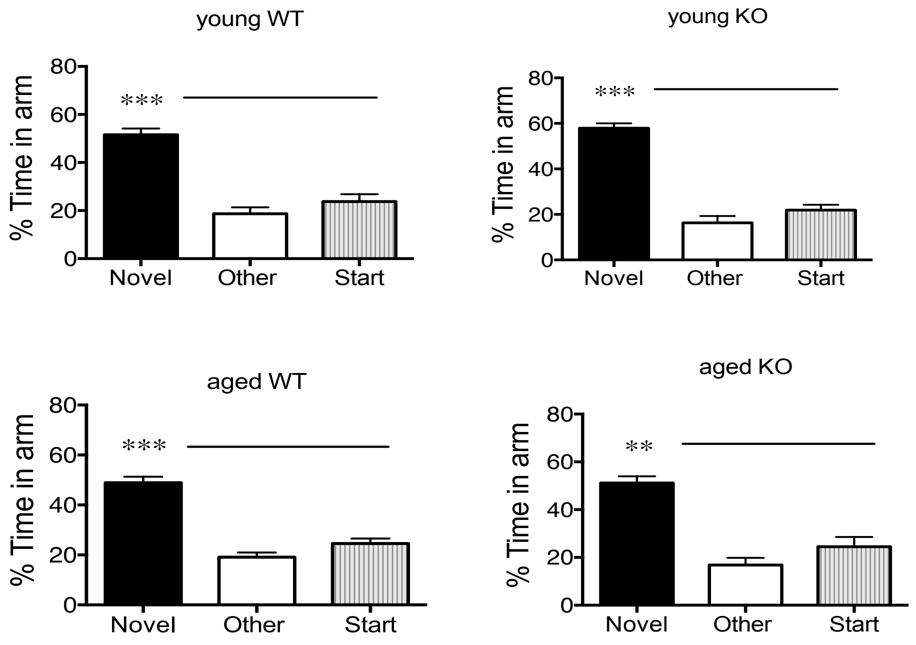

Figure S1. Initial assessment of young and aged wild-type and 11β-HSD1-deficient mice in the Y-maze following a 1 min inter-trial interval (ITI). All young (6 months, n=9/genotype) and aged (24 months, wild-type (WT) (n=13); *Hsd11b1*$^{-/-}$ (KO) (n=8)) mice showed a preference for the novel arm after a 1min ITI. *** P<0.001; **P<0.01 compared to other and start arms. Data shown are mean ± SEM.

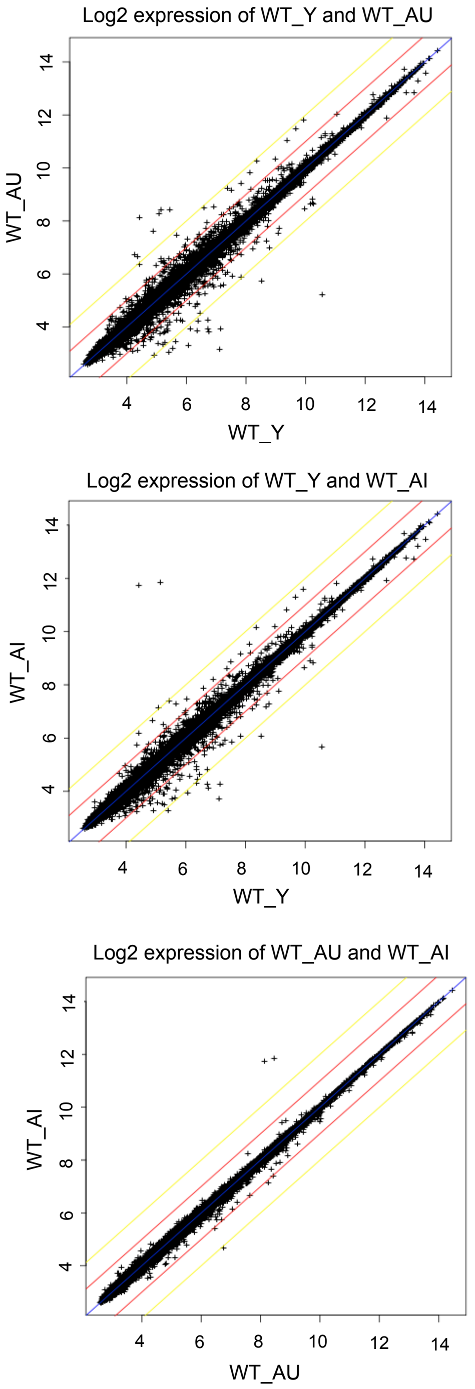

Figure S2. Comparison of scatter plots of log intensity values (four replicates combined) for young and aged wild-type mice main comparisons.
WT_AI (aged wild-type memory-impaired); WT_AU (aged wild-type memory-unimpaired); WT_Y (young wild-type). Blue line shows no change, red lines equal a 2-fold change, yellow lines are 4-fold

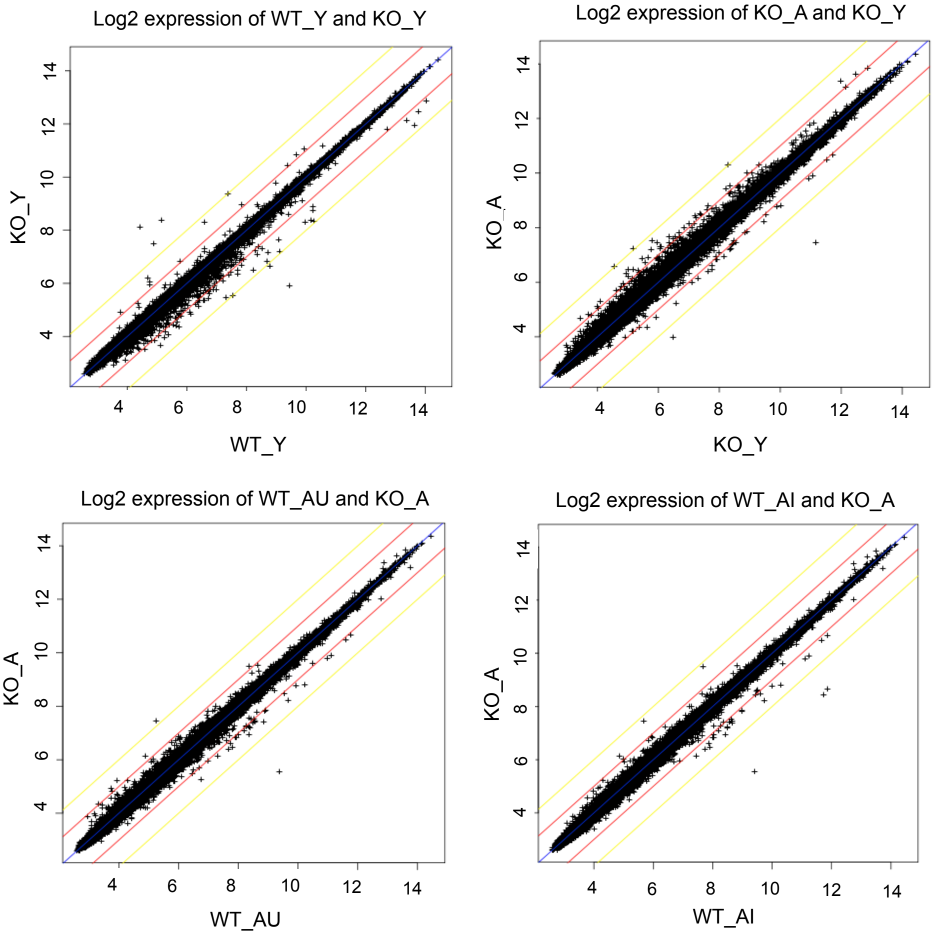

Figure S3. Comparison of scatter plots of log intensity values (four replicates combined) for young and aged wild-type and 11β-HSD1-deficient mice main comparisons.
WT_AI (aged wild-type memory-impaired); WT_AU (aged wild-type memory-unimpaired); WT_Y (young wild-type); KO_Y (young *Hsd11b1*$^{-/-}$); KO_A (aged *Hsd11b1*$^{-/-}$)
Blue line shows no change, red lines equal a 2-fold change, yellow lines are 4-fold

Supplementary Table S1

Hippocampal genes up-regulated with ageing in wild-type and 11β-HSD1-deficient mice but not affected by genotype. Gene expression level fold changes shown are from comparisons of aged (24 months) wild-type (WT_A) mice (combined WT_AU and WT_AI mice) with corresponding young (6 months) wild-type (WT) or *Hsd11b1*$^{-/-}$ (KO) mice. *, P<0.05, **P<0.01, RP scores

Affymetrix ID	Gene symbol	Gene title	Fold change WT	Fold change KO
**1426508_at**	*Gfap*	glial fibrillary acidic protein	1.6 *	2 **
**1449401_at**	*C1qc*	complement component 1, q subcomponent, C chain	1.6 *	1.7 **
**1417063_at**	*C1qb*	complement component 1, q subcomponent, beta polypeptide	1.5 *	1.6 **
**1419100_at**	*Serpina3n*	serine (or cysteine) peptidase inhibitor, clade A, member 3N	2.0 *	1.8 *
**1449254_at**	*Spp1*	secreted phosphoprotein 1	1.7 *	1.6 *
**1427371_at**	*Abca8a*	ATP-binding cassette, sub-family A (ABC1), member 8a	1.6 **	1.8 **
**1452428_a_at**	*B2m*	beta-2 microglobulin	1.5 *	1.6 *
**1418204_s_at**	*Aif1*	allograft inflammatory factor 1	1.5 *	1.7 **
**1435477_s_at**	*Fcgr2b*	Fc receptor, IgG, low affinity IIb	1.9 *	2.0 **
**1444564_at**	*Apod*	apolipoprotein D	2.0 *	2.0 **
**1450641_at**	*Vim*	Vimentin	1.6 *	1.8 **
**1422640_at**	*Pcdhb9*	protocadherin beta 9	2.0 **	1.9 **
**1454268_a_at**	*Cyba*	cytochrome b-245, alpha polypeptide	1.6 *	1.8 **
**1448620_at**	*Fcgr3*	Fc receptor, IgG, low affinity III	1.5 *	1.7 **
**1460330_at**	*Anxa3*	annexin A3	1.4 *	1.6 **
**1421792_s_at**	*Trem2*	triggering receptor expressed on myeloid cells 2	1.5 **	1.5 *
**1418580_at**	*Rtp4*	receptor transporter protein 4	2.0 *	2.0 **
**1443745_s_at**	*Dmp1*	dentin matrix protein 1	1.6 *	1.9 **
**1423547_at**	*Lyz2*	lysozyme 2	2.9 **	2.4 *
**1427076_at**	*Mpeg1*	macrophage expressed gene 1	1.6 *	2.0 **
**1450792_at**	*Tyrobp*	TYRO protein tyrosine kinase binding protein	1.6 *	1.8 **
**1434342_at**	*S100b*	S100 protein, beta polypeptide, neural	1.2 *	1.7 **
